# Potassium ion pre-intercalated MnO_2_ for aqueous multivalent ion batteries

**DOI:** 10.1007/s12200-023-00093-0

**Published:** 2023-12-01

**Authors:** Zikang Xu, Ruiqi Ren, Hang Ren, Jingyuan Zhang, Jinyao Yang, Jiawen Qiu, Yizhou Zhang, Guoyin Zhu, Liang Huang, Shengyang Dong

**Affiliations:** 1 School of Environmental Science and Engineering, School of Chemistry and Materials Science, Nanjing University of Information Science and Technology, Nanjing, 210044 China; 2 Wuhan National Laboratory for Optoelectronics, School of Optical and Electronic Information, Huazhong University of Science and Technology, Wuhan, 430074 China

**Keywords:** Aqueous batteries, Multivalent ion batteries, Magnesium ion, Aluminum ion, MnO_2_

## Abstract

Manganese dioxide (MnO_2_), as a cathode material for multivalent ion (such as Mg^2+^ and Al^3+^) storage, is investigated due to its high initial capacity. However, during multivalent ion insertion/extraction, the crystal structure of MnO_2_ partially collapses, leading to fast capacity decay in few charge/discharge cycles. Here, through pre-intercalating potassium-ion (K^+^) into δ-MnO_2_, we synthesize a potassium ion pre-intercalated MnO_2_, K_0.21_MnO_2_·0.31H_2_O (KMO), as a reliable cathode material for multivalent ion batteries. The as-prepared KMO exhibits a high reversible capacity of 185 mAh/g at 1 A/g, with considerable rate performance and improved cycling stability in 1 mol/L MgSO_4_ electrolyte. In addition, we observe that aluminum-ion (Al^3+^) can also insert into a KMO cathode. This work provides a valid method for modification of manganese-based oxides for aqueous multivalent ion batteries.

**Graphical Abstract:**

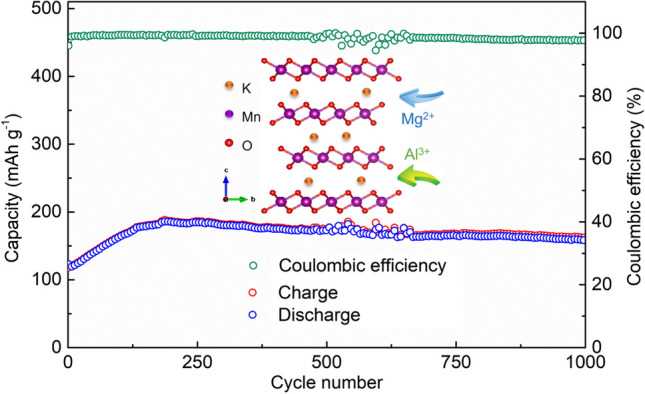

**Supplementary Information:**

The online version contains supplementary material available at 10.1007/s12200-023-00093-0.

## Introduction

Li-ion batteries (LIBs) have penetrated all aspects of the society, in portable electronics, electric mobility equipment, and even in large-scale energy storage systems [1–5]. Unluckily, with the shortage of lithium resources, the utilization of LIBs will be hindered by the rising price in the future. This problem has stimulated the investigation of promising alternatives. Due to the merits of low cost, low installation requirements, and high-level safety, aqueous rechargeable batteries (ARBs) offer an ideal option for dealing with future energy-demand pressure [6–9]. While relatively low energy density is one of the main issues of ARBSs [10–12], pairing multivalent ion carriers and exploiting high capacity cathode materials provide effective strategies to conquer the problem [13–16]. Unlike mono-valent carriers, such as Na^+ ^and K^+^, multivalent cations have the ability to transfer more than one electron, and thereby to potentially provide better energy storage. To date, ARBs based on multivalent cations, for example Zn^2+^, Mg^2+^, Ca^2+^, and Al^3+^, have received a lot of attention [17–20]. In particular, various effective strategies have been put forward to optimize the electrochemical behavior of Zn^2+^ storage. These approaches include the perfection of cathode materials, such as porous and tunable MOFs [21], and anode modifications [22]. Very recently, Zhou’s group proposed in situ preparation of a multi-layer electro-cross-linked electrolyte [23]. Based on such electrolyte, the assembled Zn/Zn–Alg-5/MnO_2_ full cell not only provides outstanding electrochemical performances but offers potential for practical application. However, the field of aqueous magnesium-ion batteries (MIBs) still suffers from inadequate research despite the batteries’ unique advantages.

Mg is the fifth most abundant metal element in the Earth’s crust [24–26], making it a cost-effective material for scale application. However, sluggish kinetics of divalent ions in electrode materials, caused by strong electrostatic interactions between Mg^2+^ and anions in a host framework, induces a high overpotential and a low degree of magnesiation [27, 28]. In recent years, Chevrel phase Mo_6_S_8_ [29], MnO_2_ [30] and layered V_2_O_5_ [31] have been explored for Mg^2+^ storage. Among these candidates, MnO_2_ has received a lot of attention due to its high theoretical capacity, readily accessible, low cost and environmental compatibility [32]. Up to date, variant phases of MnO_2_, including hollandite α-MnO_2_ [33, 34], spinel λ-MnO_2_ [35], and birnessite δ-MnO_2_ [36], have been studied and the research has obtained remarkable progress.

Nevertheless, serious capacity decay of MnO_2_ cycling in aqueous electrolytes is frequently observed. In recent years, employing a pre-intercalation strategy to enhance electrochemical behavior and stabilize structure integrity has been proven to be an effective method [37]. As reported by Mai’s group, the structural stability of layered vanadium oxide for Mg^2+^ storage can be improved through alkali ion pre-intercalating [38]. Thus, modifying the structure of MnO_2_ to realize more stable insertion/extraction of Mg^2+^ and obtain considerable reversible capacity is an urgent priority.

Herein, we report a potassium ion (K^+^) pre-intercalated K_0.21_MnO_2_·0.31H_2_O (KMO) as a cathode material for Mg^2+^ hosting. Through a simple sol–gel process, K^+^ is pre-intercalated into δ-MnO_2_, and the layered framework is stabilized, realizing reversible insertion/extraction of Mg^2+^. The KMO cathode in this study delivered a high specific capacity of 163 mAh/g at 0.1 A/g, satisfying rate performance, and improved long-term cycling stability. Additionally, KMO exhibits capability of aluminum ion (Al^3+^) storage, implying potential application in aqueous aluminum-ion batteries (AIBs) even though further modifications are still required.

## Results and discussion

**Fig. 1:**
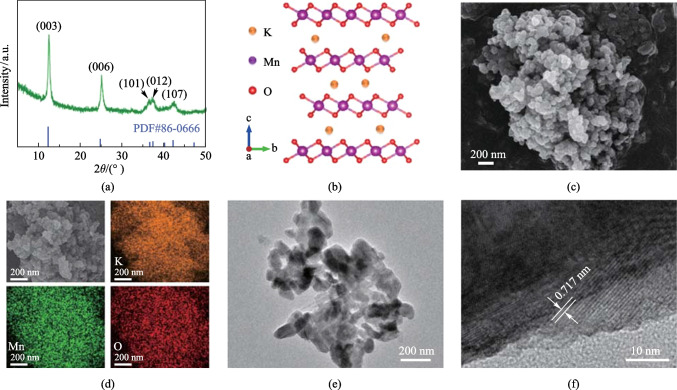
Structural characterizations of KMO. **a** XRD pattern. **b** Crystal structure schematic of KMO. **c** SEM image. **d** Elemental mapping. **e**, **f** TEM images

The crystalline structure of KMO was firstly characterized by powder X-ray diffraction (XRD). As displayed in Fig. 1a, the diffraction pattern shows the diffraction peaks of K_0.27_MnO_2_·0.54H_2_O (JCPDS No. 86-0666), which builds up by layers of edge-shared MnO_6_ (Fig. 1b). The scanning electron microscopy (SEM) image (in Fig. 1c) demonstrate that KMO possesses a nanoparticle morphology with a size of 50–100 nm. The elemental energy-dispersive X-ray (EDX) spectroscopy mapping using SEM shows that the distribution of various elements including K, Mn, and O are uniform (Fig. 1d). Besides, according to the elemental mapping, the atomic ratio of K and Mn in KMO is about 0.23 (Table S1). The morphology of nanoparticles can be further revealed by transmission electron microscopy (TEM) imagery (Fig. 1e). Meanwhile, the interplanar distance of 0.71 nm (Fig. 1f) demonstrates the (003) crystal plane of KMO. According to the data of inductively coupled plasma optical emission spectrometer (ICP-OES; can be find in Table S2) and thermogravimetric (TG) analysis (Fig. S1), the speculated formula of the as-prepared KMO is K_0.21_MnO_2_·0.31H_2_O.

**Fig. 2:**
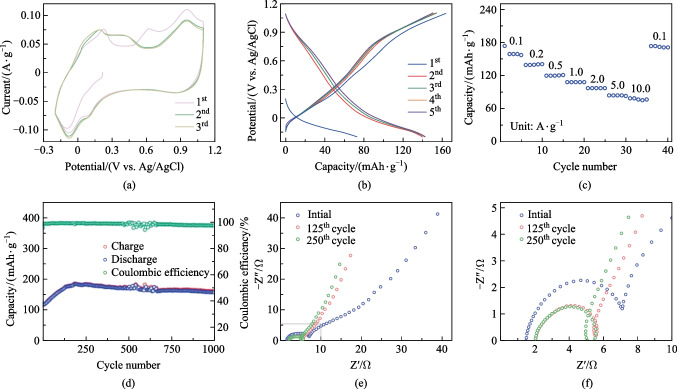
Electrochemical behaviors of KMO. **a** CV profiles at 0.2 mV/s. **b** First five GCD curves at 0.1 A/g. **c** Rate performance from 0.1 to 10.0 A/g. **d** Long-term cycling stability at 1 A/g. **e**, **f** EIS plots at different cycles

The electrochemical performances of KMO were evaluated in three-electrode cells, with the potential window limited from − 0.2 to  1.1 V, and 1 mol/L MgSO_4_ solution as the electrolyte (All potentials below are relative to those for Ag/AgCl.). As shown in Fig. 2a, the cyclic voltammetry (CV) curves almost overlap after the first cycle, which hints at the high electrochemical reversibility of KMO regarding magnesium ion storage. Notably, in the first five galvanostatic charge–discharge (GCD) profiles (Fig. 2b), KMO electrode provides approximately 163 mAh/g charge capacity at the current density of 0.1 A/g without obvious capacity degradation. In addition, when raising the current density from 0.1 to 10 A/g, the electrode exhibits a benign rate performance with approximately 78 mAh/g at 10 A/g. It is worth noting that the capacity slightly improves, compared to that of the initial value at 0.1 A/g, when the current density returns to 0.1 A/g again, which may arise from an activation process. Such a phenomenon also happened when carrying out the long-term cycling test, with the capacity increasing from 110 mAh/g to the highest capacity of 185 mAh/g at the high current density of 1 A/g. What’s more, the KMO delivered favorable cycling performance of Mg^2+^ storage, remaining about 86.7% capacity retention of the maximum over 1000 cycles, exceeding the majority of MnO_2_ cathode materials that have been reported [27, 34, 39]. Further, electrochemical impedance spectroscopy (EIS) was conducted to check the activation process. Figure 2e shows that after 125th and 250th cycles, and relative to pristine, the KMO cathode shows a smaller semicircle in the high-frequency zone and a line with a larger slope in the low-frequency zone than initial, implying a smaller charge-transfer resistance and faster Mg^2+^ diffusion kinetics after the cycling process. Such results further prove the capacity increase and activation process during cycling.

**Fig. 3:**
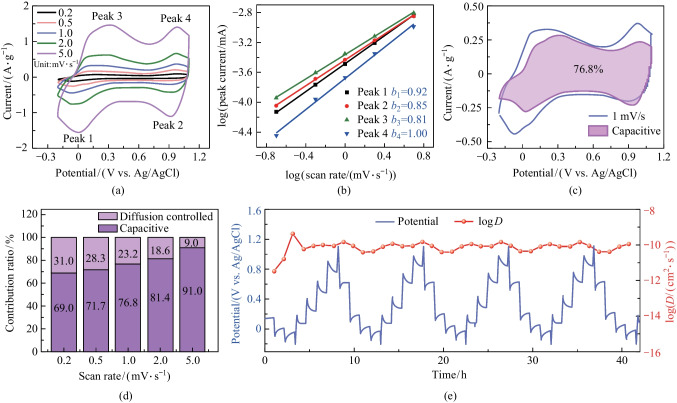
Kinetic analysis of KMO electrode. **a** CV curves at different scan rates. **b** log(*i*) vs. log(*v*) plots of reduction/oxidation currents response. **c** Capacitive contribution at 1 mV/s. **d** Contribution ratio of diffusion-dominated capacities and non-diffusion-dominated capacities at different scan rates. **e** GCD profiles with the GITT test (0.1 A/g for 10 min and then 1 h rest) and the corresponding diffusion coefficient

Furthermore, a cyclic voltammetry (CV) test under different scan rates was carried out to evaluate the electrochemical kinetics of a KMO electrode for Mg^2+^ storage. As depicted in Fig. 3a, during the increase of scan rates, the reduction/oxidation peak currents become apparently increased, and the shapes of CV curves show good agreement, indicating the excellent electrochemical reversibility of the KMO electrode. Typically, the stored charge originates from two parts: diffusion-dominated process and non-diffusion-dominated capacitive process, and the capacity contribution can be calculated by the power-law equation [40]:

1 $$i=a{v}^{b},$$

where *i* represents the peak currents, *a* and *b* represent coefficients, and *v* signals the scan rate. Additionally, the *b* value is used to assess the capacity domination. When *b* is 0.5, the capacity is determined by the diffusion-controlled behavior, while *b* = 1 indicates the capacitive process.

Equation (1) can be further transformed to the formulation:

2 $$\mathrm{log}\left(i\right)=\mathrm{log}\left(a\right)+b\mathrm{log}\left(v\right).$$

Thus, the values of *b* can be calculated from the slopes of log(*i*) vs. log(*v*). Moreover, the specific contribution of the two processes can be calculated through Eq. (3) [41]:

3 $$i={k}_{1}v+{k}_{2}{v}^{1/2},$$

where *k*_1_*v* represents the capacitive-type contribution, while *k*_2_*v*^1/2^ denotes the diffusion-controlled counterpart. As shown in Fig. 3b, the *b* values vary from 0.8 to 1, indicating that the capacity is controlled by a combination of the both of the processes. Meanwhile, the contribution of capacitive-controlled storage gradually increases when increasing the scan rates from 0.2 to 5 mV/s, and the proportion of capacitive storage finally comes to 91% at 5 mV/s (Fig. 3c, d and Fig. S2). To further evaluate the diffusion kinetics of Mg^2+^ in the KMO electrode, the galvanostatic intermittent titration technique (GITT) was implemented. The result, presented in Fig. 3e, shows that the calculated diffusion coefficient is between 10^−10^ and 10^−9^ cm^2^/s, indicating fast Mg^2+^ conduction in KMO [42–45].

*Ex-situ* XRD was conducted to investigate the structure evolution of KMO during the Mg^2+^ storage process. The first cycle of the GCD profile and corresponding XRD patterns are described in Fig. S3a and S3b. No new diffraction peaks are detected during insertion/extraction of Mg^2+^, demonstrating that KMO maintains a consistent layered structure. As displayed, the (003) peak (enlarged in Fig. S3c) shifts slightly to a higher 2*θ* angles during the discharge process, suggesting the decrease of the corresponding interlayer spacing. After a full charge/discharge cycle, the behavior of the (003) plane shows subtle deviation, demonstrating a reversible insertion/extraction of Mg^2+^. According to the data of ICP-OES, shown in Table S3, the content of Mg^2+^ in the electrolyte increases from the first uncharged to charged state, implying the existence of Mg^2+^ extraction from electrode. Meanwhile, the concentration of K^+^ slightly increases, which may arise from minor co-extraction of K^+^ along with Mg^2+^. In addition, the dissolution problem of manganese in MnO_2_ can be effectively inhibited.

We also compared the electrochemical behaviors of the KMO in 1 mol/L ZnSO_4_ aqueous electrolyte. As shown in Fig. S4a, a couple of redox peaks at around 0.42 and 0.58 V can be found at 1 mV/s. Fast capacity loss and unfavorable rate capability also happened for Zn^2+^ storage (Fig. S4b and S4c). For example, only 37.2 mAh/g could be retained at 2 A/g. Besides, KMO suffers a fast capacity loss in the initial cycles (Fig. S4d).

We selected VO_2_ (Fig. S5a, monoclinic VO_2_ (B) phase) as an anode and fabricated a full cell with a KMO cathode. Briefly, a VO_2_ anode could provide a reversible capacity of about 150 mAh/g at a current density of 0.1 A/g (Fig. S5b). As shown in Fig. S5c, when the current density was increased to 1 A/g, the capacity of 41.1 mAh/g could be retained. A capacity retention of 80.9% could be obtained after 300 cycles at 1 A/g. The assembled KMO||VO_2_ full cell could deliver about 80 mAh/g based on the active mass of cathode at 0.1 A/g (Fig. S6a). When the current density was elevated to 1 A/g, the corresponding capacity was about 20 mAh/g (Figure S6b). Besides, the capacity retention wass about 58.0% after 100 cycles.

**Fig. 4:**
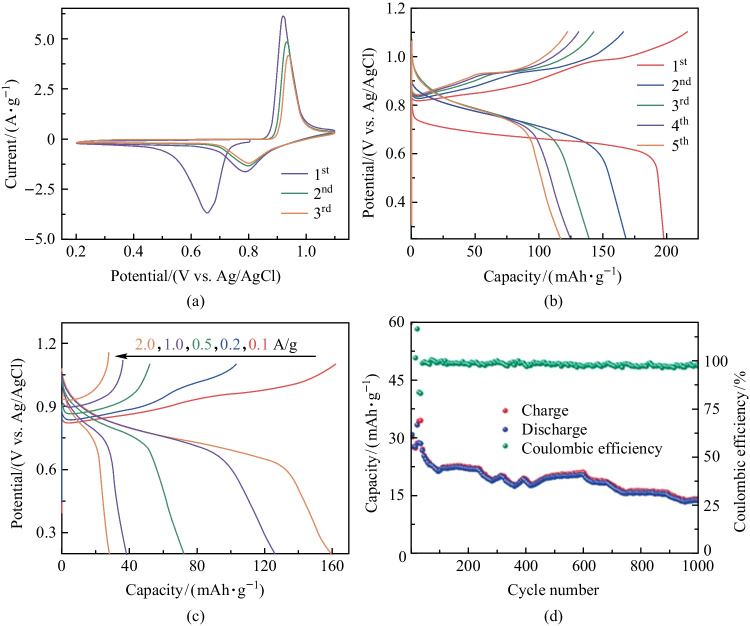
Electrochemical performance of KMO electrode for Al^3+^ storage. **a** CV curves at 1 mV/s. **b** First five GCD curves at 0.1 A/g in 0.5 mol/L Al_2_(SO_4_)_3_ electrolyte. **c** Rate performance. **d** Cycling stability at 0.5 A/g

Since divalent Mg^2+^ can effectively insert into the KMO electrode, we suspected that Al^3+^ can also insert into KMO. We conducted routine electrochemical measurements to test this. Figure 4a shows the first three CV curves with a pair of distinct redox peaks at around 0.80 and 0.93 V. Typically, from the first discharge profile in Fig. 4b, an obvious discharge plateau can be detected, which may result from the structure reconfiguration. As is well-acknowledged, the inserted Al^3+^ ions generally possess relatively high electrostatic interaction with the host materials, and thus causes structural collapse, leading to fast capacity decay and poor cycling stability [46–48]. Such a disadvantage applies to the KMO electrode as well, which can be detected from the GCD curves. After five cycles, the specific charge capacity quickly faded from 200 to 125 mAh/g. As shown in Fig. 4c, only 28.3 mAh/g could be obtained at 2 A/g. On the other hand, poor cycling capacity (capacity retention of 45.1% after 1000 cycles at 0.5 A/g) also creates a requirement for more in-depth modification of KMO structure. KMO has the ability to store Al^3+^ in aqueous electrolyte, but further investigation, for all potential electrode materials and electrolytes, is needed.

## Conclusions

In summary, through pre-intercalating potassium ions into manganese dioxide, we explored an effective cathode material for Mg^2+^ storage. The KMO electrode can deliver a considerable capacity of 185 mAh/g at a current density of 1 A/g in 1 mol/L MgSO_4_ aqueous solution. In addition, after an activation process, the electrochemical impedance greatly decreases and the layered-structure KMO material exhibits improved rate capability (78 mAh/g at a current density of 10 A/g) and long-term cycling stability (capacity retention 86.7% over 1000 cycles). Moreover, Al^3+^ can also be inserted into the KMO host, but the structure transforms during Al^3+^ insertion/extraction. Further investigation is required to improve electrochemical performance. Finally, we believe that this work promotes the study of cathode materials for Mg^2+^/Al^3+^ storage and offers an insight to modification of metal-oxide cathodes for non-monovalent storage.

### Supplementary Information

Below is the link to the electronic supplementary material.

Supplementary file1 (PDF 555 KB)

## Data Availability

The data that support the findings of this study are available from the corresponding author, upon reasonable request.
