# Supplementary material for: Potassium ion pre-intercalated MnO2 for aqueous multivalent ion batteries
Source: Front Optoelectron. 2023 Dec 1;16(1):39. doi: 10.1007/s12200-023-00093-0 (PMC10692024; doi:10.1007/s12200-023-00093-0)
Supplement: Supplementary file 1 — Supplementary file1 (PDF 555 KB) [file 12200_2023_93_MOESM1_ESM.pdf]

Supplementary Information

Potassium ion pre-intercalated MnO_2 for aqueous multivalent ion batteries

Zikang Xu[1] · Ruiqi Ren[1] · Hang Ren[1] · Jingyuan Zhang[1] · Jinyao Yang[1] · Jiawen Qiu[1] · Yizhou Zhang[1] · Guoyin Zhu[1, *] ·Liang Huang[2, *] · Shengyang Dong[1, *]

[1]School of Environmental Science and Engineering, School of Chemistry and Materials Science, Nanjing University of Information Science & Technology, Nanjing 210044, China

E-mail: dongsyst@nuist.edu.cn (S. Dong); gyzhu@nuist.edu.cn (G. Zhu)

[2]Wuhan National Laboratory for Optoelectronics, School of Optical and Electronic Information, Huazhong University of Science and Technology, Wuhan 430074, China

E-mail: huangliang421@hust.edu.cn

1. Methods

1.1. Preparation of KMO

Potassium ion pre-intercalated MnO_2 (KMO) was synthesized by a sol-gel process based on the previous report with a minor modification [1]. Typically, 1.5 g $KMnO_4$ was dissolved in 25 mL deionized (DI) water to obtain solution A. 2.5 g D(+)-Glucose was dissolved into 10 mL DI water to obtain solution B. Then solution A was quickly added into solution B with vigorous stirring for 15 seconds. The mixed solution was allowed to stand until a reddish sol was formed and readily turned into a brown gel in 30 seconds. The brown gel was allowed to cool down for 30 minutes before it was moved to an oven and dried at 110 °C for 12 hours. The obtained dark brown product was then ground and washed with DI water and ethanol for several times, respectively. After being dried in vacuum at 60 °C overnight, the final solid KMO product was obtained.

1.2. Preparation of VO_2

The VO_2 was synthesized by a facile hydrothermal method. Typically, the V_2O_5 powder (0.3638 g) and $H_2C_2O_4$ powder (0.5402 g) were dissolved into deionized water (8 mL) by constant magnetic stirring for about 1 h at 75 °C until the solution turns dark blue. Then 20 mL 3 % H_2O_2 was added to the above solution and kept stirring for about 20 minutes. After that, the mixture was transferred into a 50 mL autoclave with a Teflon liner and kept at 180 °C for 10 h. Finally, the precipitate was collected by centrifuging and washing with deionized water and ethanol several times, and then dried at 60 °C for 12 h under a vacuum.

1.3. Structure characterization

X-ray diffraction (XRD) patterns were obtained on Rigaku Ultra 250 with Cu Kα radiation (1.5406 Å, 40 kV 40 mA). The morphologies and microstructures were collected with scanning electron microscopy (SEM, ZEISS, Gemini300S), and transmission electron microscopy (TEM, JEOL, JEM-2100F). Thermogravimetric analysis (TGA) was done on a TA STD650 thermal analyzer at a temperature ramp of 5 °C min^{-1}. The chemical composition was determined by inductively coupled plasma optical emission spectrometer (ICP-OES, Perkin Elmer 2100 DV avio200).

1.4. Electrochemical characterization

All the electrochemical tests were performed in a three-electrode cell. To prepare KMO working electrode, 70 wt.% KMO, 20 wt.% acetylene black and 10 wt.% carboxymethyl cellulose (CMC) and styrene-butadiene rubber (SBR) (the mass ratio of CMC/SBR is 2:1) binder were mixed and dispersed into deionized water to form a uniform slurry. Then the slurry was coated onto 10 mm diameter carbon paper, followed by drying in vacuum at 80 °C for 12h. The mass loading of KMO working electrode is about 1.5 mg cm^{-2}. The VO_2 electrode was prepared through the same method. The counter electrode is activated carbon (AC) films, prepared by mixing AC, acetylene black and polytetrafluoethylene (PTFE) in a mass ratio of 8:1:1. Ag/AgCl electrode is chosen as the reference electrode. As for ex situ XRD test, KMO free-standing film electrode was also prepared as the working electrodes, which employed PTFE as the binder.

The cyclic voltammetry (CV) and electrochemical impedance spectroscopy (EIS) were tested on a CorrTest CS2350H electrochemical workstation. The galvanostatic charge-discharge (GCD) were carried out on LANBTS cell test system with the potential windows of -0.2 ~ 1.1 V, -0.9 ~ 0 V for KMO and VO_2 in 1 M $MgSO_4$ electrolyte, respectively, and 0.2 ~1.1 V in 0.5 M $Al_2(SO_4)_3$ electrolyte.

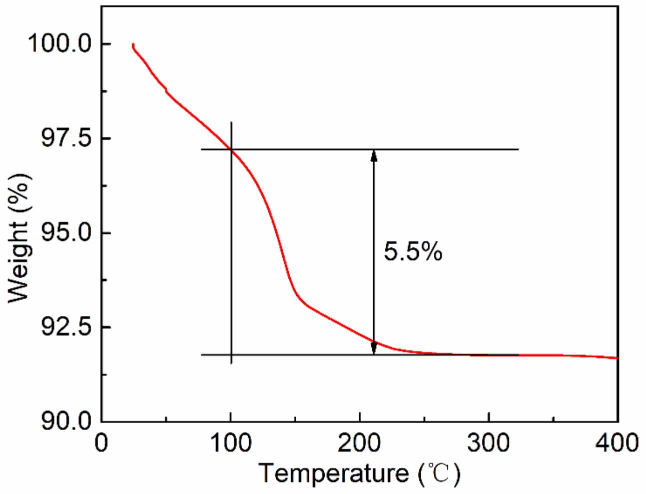

Fig. S1 TG curve of KMO.

As the molar mass of $K_{0.23}MnO_2$ is certain, the content of crystal water can be calculated from the equation: $\frac{m(H_2O)}{m(KMO)} = \frac{n{*}M(H_2O)}{M(KMO)}$, where m and M present mass and molar mass, n present the content of crystal water.

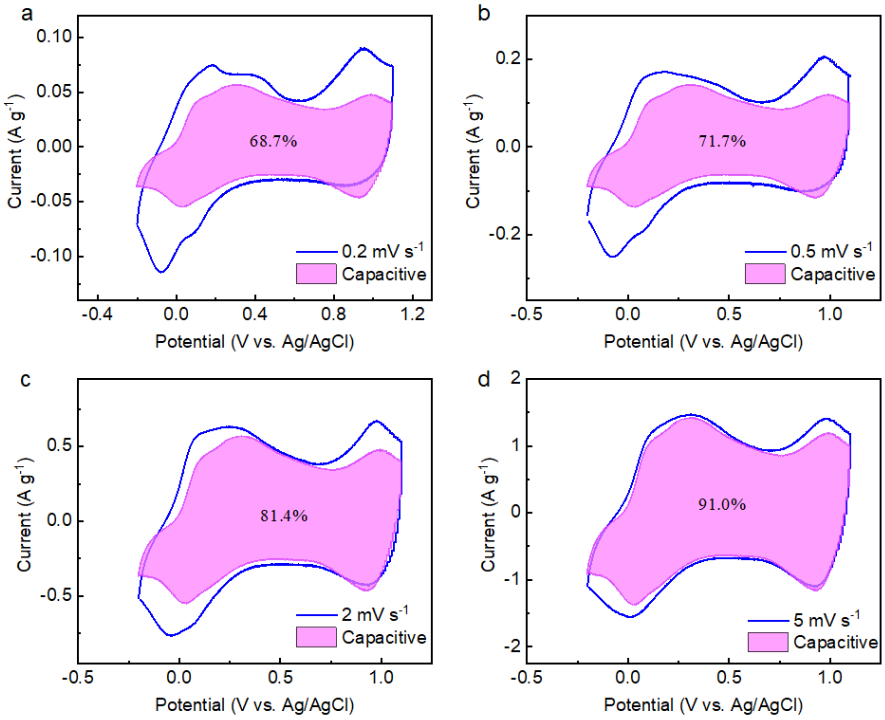

Fig. S2 Capacitive contribution at (a) 1 mV s^{-1}, (b) 0.5 mV s^{-1}, (c) 2 mV s^{-1} and (d) 5 mV s^{-1}.

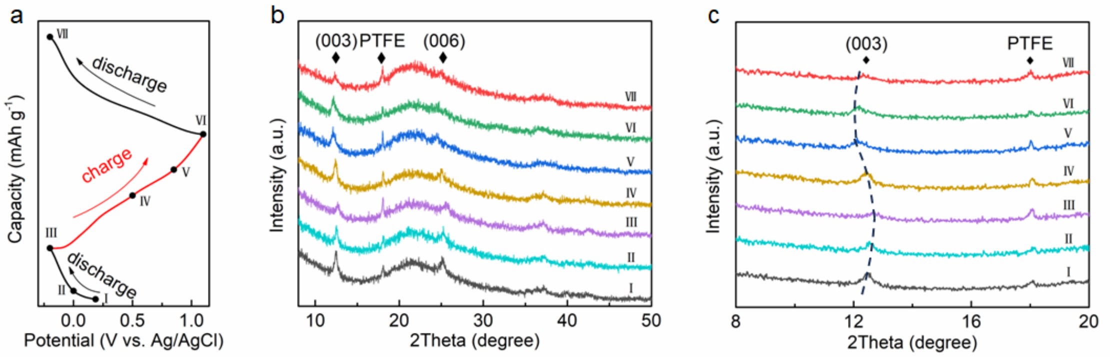

Fig. S3 (a) GCD profiles at 0.1 A g^{-1}. (b, c) Ex situ XRD patterns at different charge states.

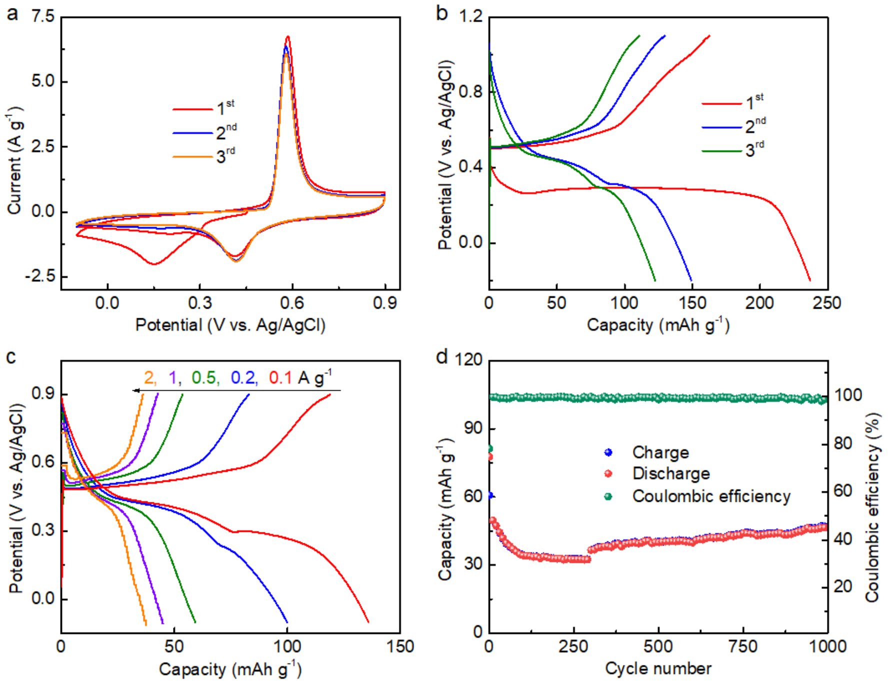

Fig. S4 (a) CV curves of KMO in 1 M $ZnSO_4$. (b) GCD profiles at 0.1 A g^{-1}. (c) Rate performances at 0.1~2 A g^{-1}. (d) Cycling stability at 1 A g^{-1}.

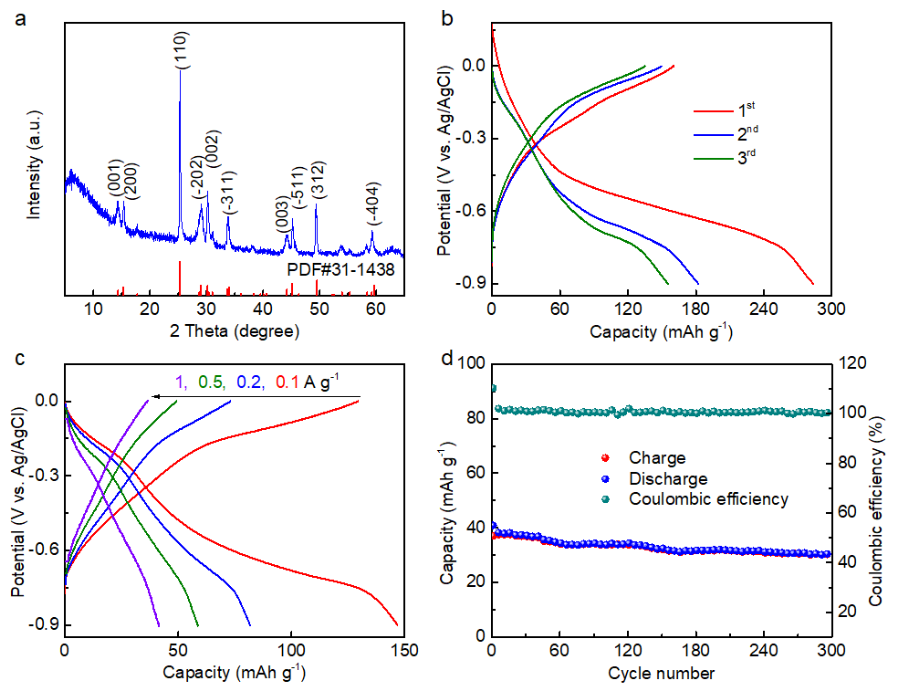

Fig. S5 (a) XRD pattern of VO_2. (b) GCD profiles of VO_2 at 0.1 A g^{-1} in 1 M $MgSO_4$. (c) Rate performance from 0.1~1 A g^{-1}. (d) Cycling stability at 1A g^{-1}.

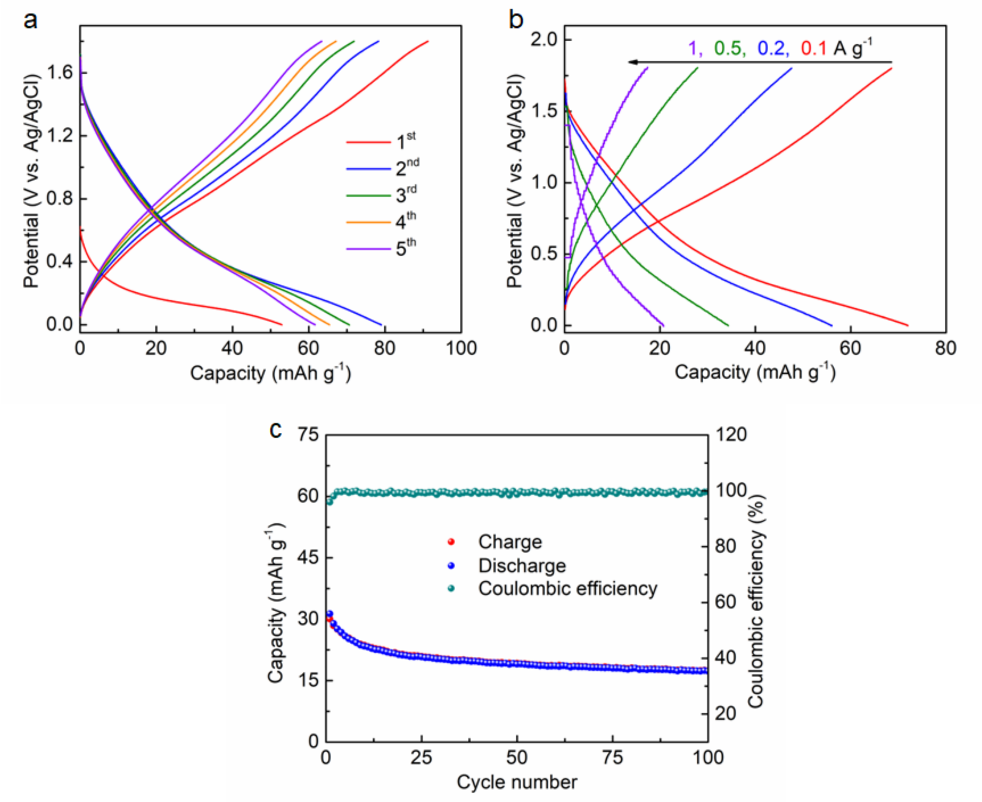

Fig. S6 (a) GCD profiles of KMO||VO_2 full cell at 0.1 A g^{-1} (based on the active mass of cathode) in 1 M $MgSO_4$. The mass ratio of KMO and VO_2 is 1:1. (b) Rate performance. (c) Cycling stability at 0.5 A g^{-1}.

Table S1 Element contents by EDS mapping.

Element	Weight fraction (%)	Atomic ratio (%)
O	39.48	67.96
Mn	52.12	26.13
K	8.4	5.91

Table S2 Element contents by ICP-OES.

Element	Sample weight (mg)	Weight fraction (%)
K	13.6	7.1
Mn	13.6	46.8

The atomic ratio of K and Mn can be calculated based on the following equation:

$$Atomic\ ratio = \frac{m_K/M_K}{m_{Mn}/M_{Mn}}$$

Where m and M denote mass and molar mass of each element, respectively.

Table S3 Element contents of electrolyte by ex situ ICP-OES.

Element	1st discharge	1st charge
K	139.2	1596
Mn	1.1	5.5
Mg	35510	42780

*Unit: mg L^{-1}

It should be noted that the errors of Mg may be due to the large dilution multiples because of its high concentration in electrolyte. However, due to the same dilution condition of Mg in discharge and charge states, the change trend of Mg is still significant.

Reference

1. Ching, S.; Petrovay, D. J.; Jorgensen, M. L.; Suib, S. L.: Sol−gel synthesis of layered birnessite-type manganese oxides. Inorg. Chem. **36**(5), 883-890 (1997).
